# Alleviation of Chronic Low Back Pain due to Bilateral Traumatic L4 Pars Interarticularis Fractures Relieved With Steroid Injections

**DOI:** 10.7759/cureus.9821

**Published:** 2020-08-17

**Authors:** Amnon A Berger, Jamal Hasoon, Ivan Urits, Omar Viswanath, Anthony Lee

**Affiliations:** 1 Department of Anesthesia, Critical Care and Pain Medicine, Beth Israel Deaconess Medical Center, Harvard Medical School, Boston, USA; 2 Pain Management, Valley Pain Consultants - Envision Physician Services, Phoenix, USA

**Keywords:** chronic pain, interventional pain therapy, pars fracture, low back pain, sports injuries

## Abstract

Chronic back pain affects 20% of the adult population in the United States and is a significant source of disability and healthcare expenditure. One of the most common causes of chronic back pain is spondylosis. These changes result from age-related degeneration of the lumbar spine. As a result of this degeneration, spondylolisthesis can develop. Spondylolysis is a fracture of the pars interarticularis. It affects younger patients and is more prevalent in adolescents and elite athletes. It can be a debilitating condition that may force athletes into retirement as well as impair them with chronic pain and disability. Traditional treatment options include conservative management such as medications, rest, physical therapy, and rehabilitation. Surgery is reserved for patients who do not respond to conservative measures. Here we present the case of a 39-year-old mixed martial arts fighter with bilateral L4 pars interarticularis fractures and chronic low back pain. After failing conservative treatment options, this patient finally obtained significant relief with steroid injections at the level of the defect. The patient continues to do well with occasional injections and is able to maintain his mixed martial arts career. This case report provides evidence that injection therapy is a feasible alternative to surgery in patients who fail conservative therapy.

## Introduction

Spondylolysis refers to a posterior fracture at the pars interarticularis. It affects 3%-10% of the population and can be a cause of low back pain (LBP). Etiology may include repetitive microtrauma of the spine as well as acute traumatic injuries. Spondylolisthesis occurs when there is translation of the vertebral bodies and can also occur with these injuries. Continued degeneration at the vertebral levels can lead to facet instability, neuroforaminal stenosis, and pressure on exiting nerve roots [1]. Unilateral acute fractures of the pars interarticularis can often heal over time. However, bilateral fractures may progress to spondylolisthesis [2]. These injuries also have an increased risk of neurological deficits and chronic pain [3].

Conservative therapy is effective in 84%-95% of patients and includes rest, discontinuation of high-impact sports, nonsteroidal anti-inflammatory drugs (NSAIDs), bracing, and physical therapy (PT) [2]. Surgery is reserved for the minority of patients who fail to respond to conservative therapy as it carries the risks of tissue injury, bleeding, infection, and hardware failure [3,4]. Injection therapy could be an option for some patients. Pain injections have the advantage of a same-day procedure visit as well as minimal risks or side effects. Radiofrequency ablation has also been described in a patient with a fracture of the pars interarticularis [5]. We describe our experience treating a mixed martial arts (MMA) fighter with bilateral fractures at the L4 pars interarticularis with steroid injections.

## Case presentation

**Figure 1:**
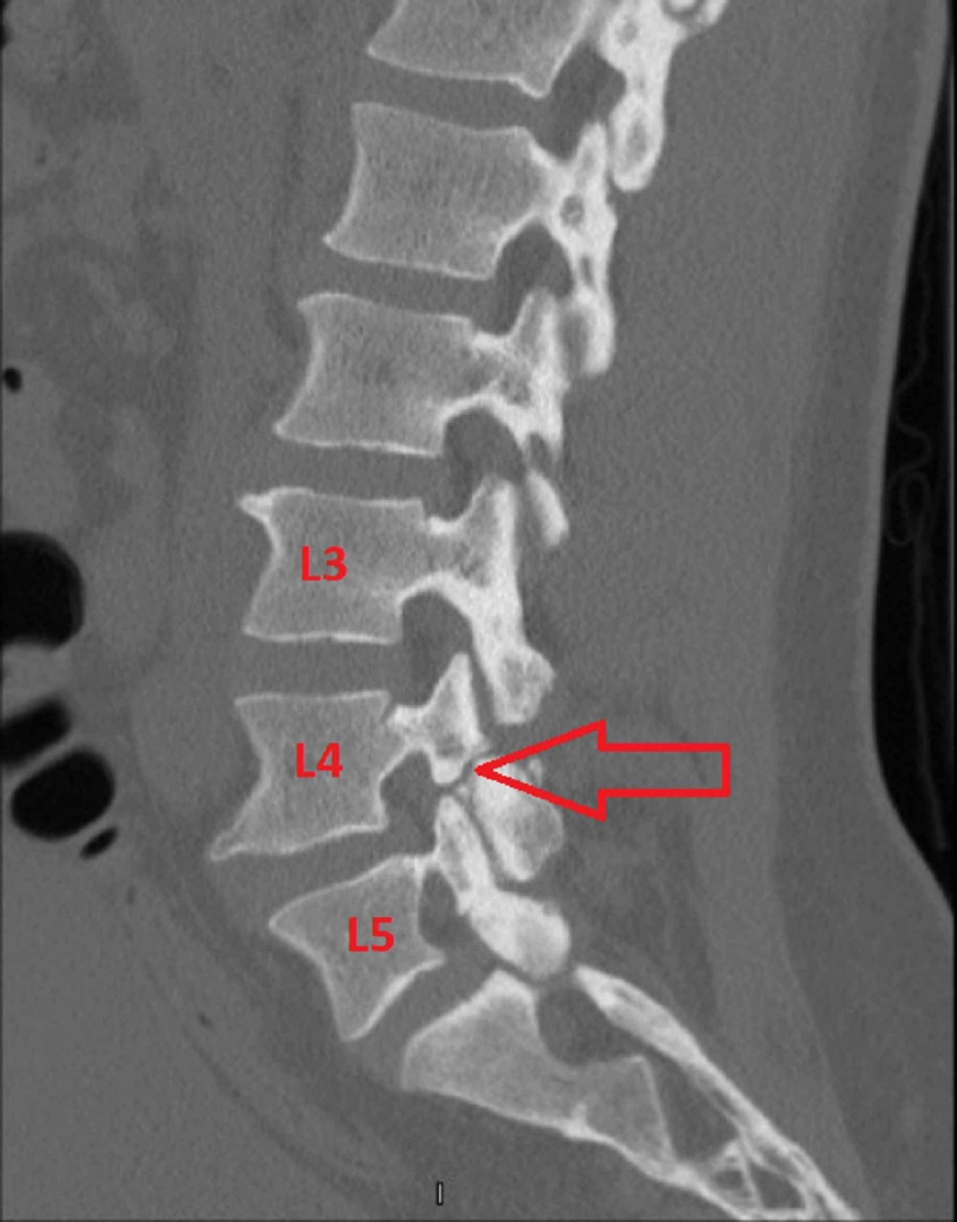
Sagittal CT of the lumbar spine Sagittal CT of the lumbar spine demonstrating a defect at the L4 level. The red arrow highlights a fracture at the L4 pars interarticularis.

The patient was a 39-year-old male who was an active MMA fighter. He had been practicing MMA since childhood and reported severe LBP for several years. He presented to the pain clinic endorsing constant LBP that occasionally radiated to the left lateral thigh. He also endorsed moderate buttock pain. The pain was stabbing in nature and worse during MMA training activities and rotational movements. The pain was rated as 9/10 intensity on a numerical rating scale (NRS). The pain drastically interfered with his ability to continue MMA activities. He underwent X-ray imaging which revealed no obvious defects. However, a CT scan of the lumbar spine demonstrated bilateral L4 pars interarticularis fractures (Figure 1). 

The patient had tried medication management with ibuprofen and acetaminophen with minimal relief. He also completed an extensive course of PT without any improvement. The patient was not interested in surgical options and was offered interventional pain injections targeting the defects. The patient was counseled regarding the risks and benefits of these injections and consented to proceed with the procedure.

**Figure 2:**
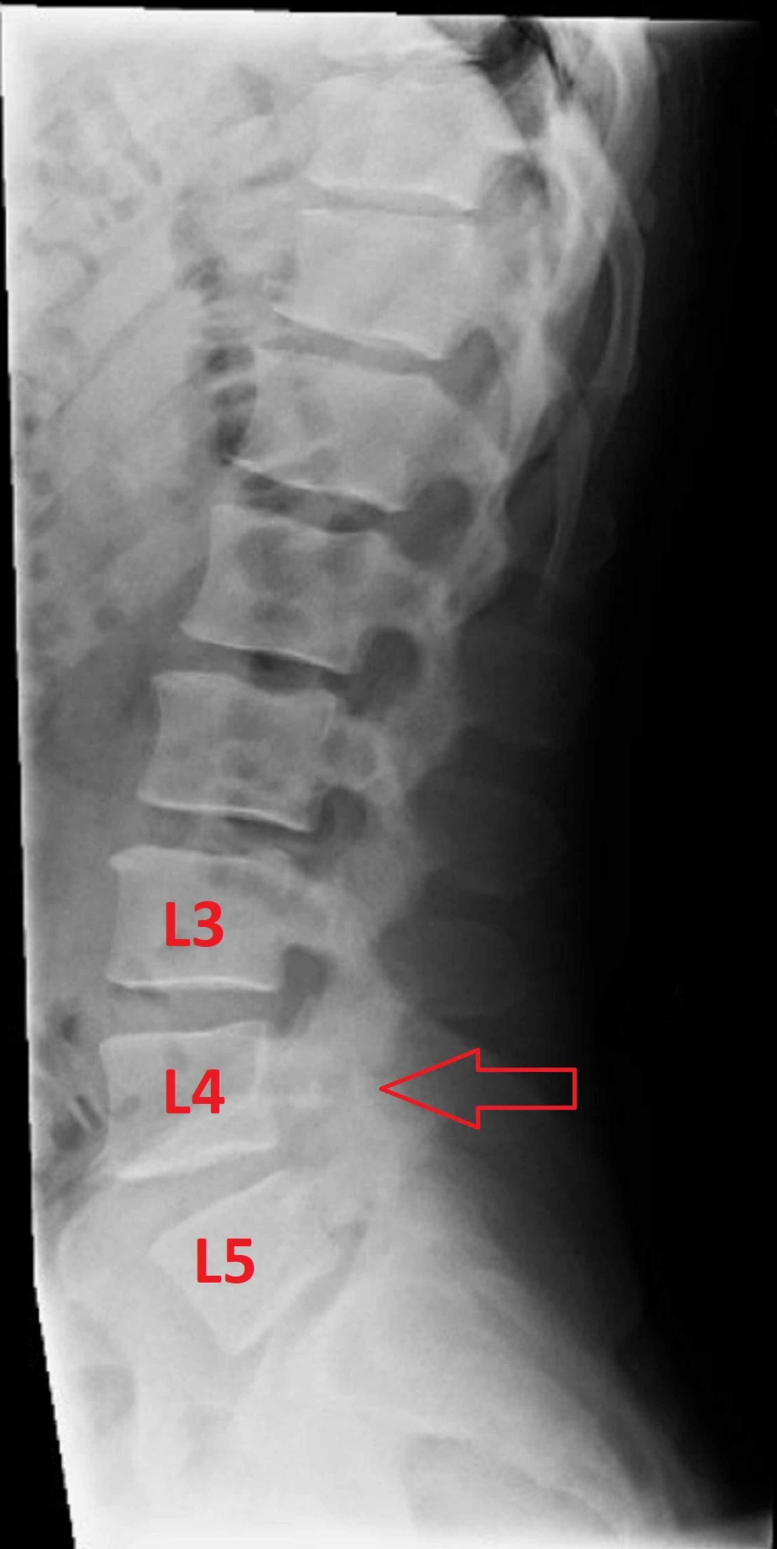
Lateral X-ray of the lumbar spine The red arrow highlights the fracture at the L4 pars interarticularis, which was the target for the injection.

**Figure 3:**
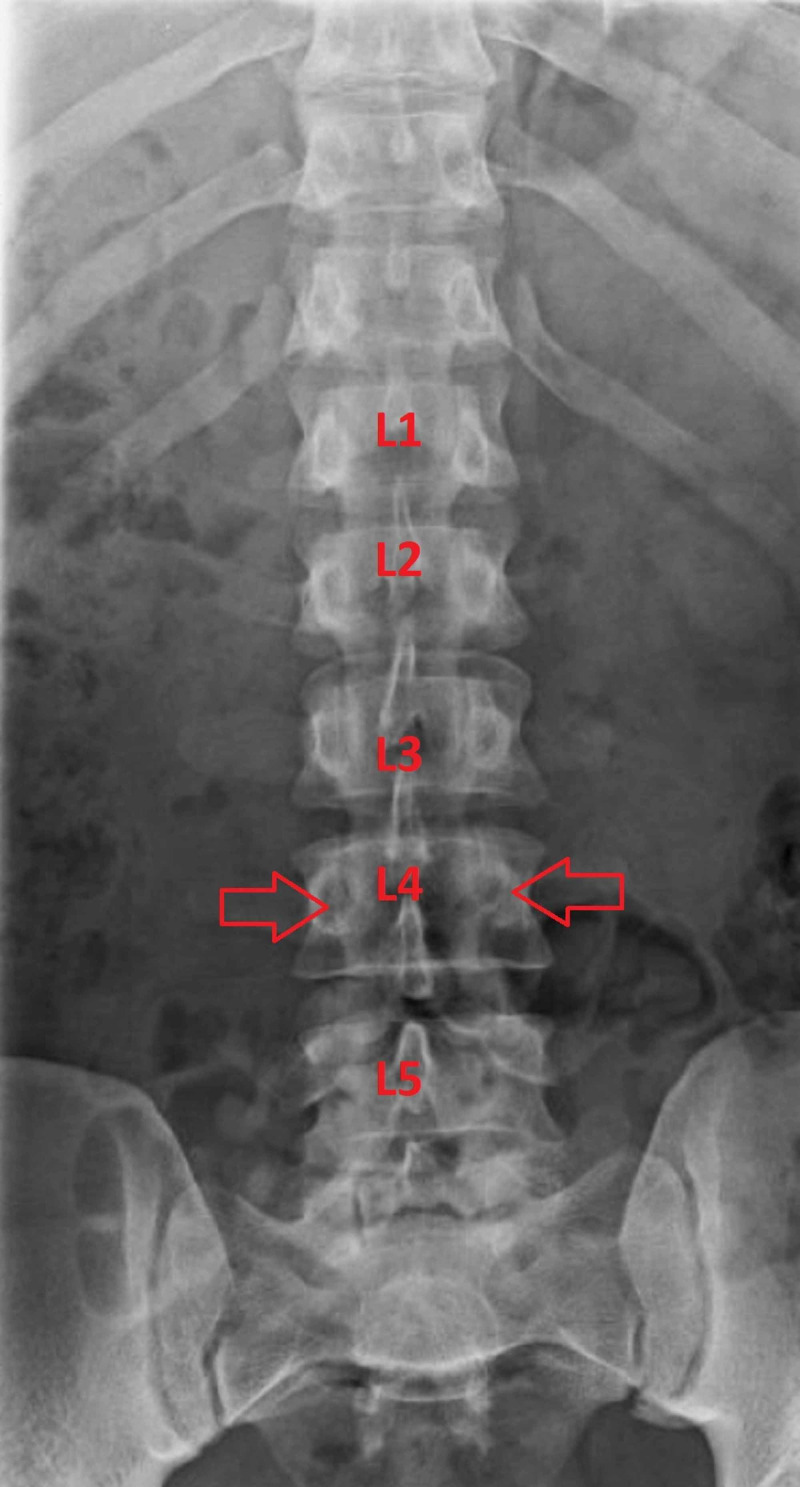
Anterior-posterior X-ray of the lumbar spine Anterior-posterior X-ray of the lumbar spine with arrows indicating the target points of the injections at the pars interarticularis near the level of the pedicle. The needles were advanced until contact with bone was made, then the medications were deposited.

He underwent bilateral injections at the L4 level targeting the pars interarticularis fractures. The solution used for the injections included 0.5 mL of 1% lidocaine mixed with 20 mg methylprednisolone per side. The blocks were performed under fluoroscopy without complications (Figures 2, 3).

## Discussion

The patient reported significant improvement of his pain shortly after the injections and noted 0/10 pain on NRS that lasted for six months. The patient underwent repeat injections at the same level after six months and again obtained significant relief. He was also able to participate fully in MMA training activities. The patient continues to get occasional steroid injections with excellent results. He has also been able to continue his MMA career. The patient has been followed for three years with successful injection treatments, no complications, and complete pain relief.

LBP causes significant morbidity and carries an estimated healthcare spending cost of $67.5 billion to $94.1 billion annually in the United States [6]. Spondylolysis affects a much smaller portion of the population but is mostly seen in young athletes, such as our patient [1-3]. Unfortunately, some of these patients can fail to respond to conservative therapy and may be forced to consider surgery or discontinue their participation in athletic activities. Our case provides encouraging evidence for treating patients suffering from spondylolysis with interventional pain injections.

## Conclusions

Spondylolysis is unique in that it more commonly occurs in younger patients and elite athletes. Patients who fail to respond to conservative treatment may be forced into a dilemma of considering surgery or discontinuing high-impact activities. Injection therapy has been used extensively for chronic pain conditions and has an established safety profile. These interventional pain procedures offer an additional option to patients suffering from spondylolysis who fail to respond to conservative therapy but are not interested in surgical management. Physicians should consider the risks and benefits of interventional pain injections for patients with spondylolysis before referral for surgical management. Additionally, future clinical trials should be considered to further investigate the effectiveness of this treatment for bilateral pars interarticularis fractures.
